# Interplay Between Non-Canonical NF-κB Signaling and Hepatitis B Virus Infection

**DOI:** 10.3389/fimmu.2021.730684

**Published:** 2021-09-29

**Authors:** Xinyu Lu, Qianhui Chen, Hongyan Liu, Xiaoyong Zhang

**Affiliations:** State Key Laboratory of Organ Failure Research, Guangdong Provincial Key Laboratory of Viral Hepatitis Research, Department of Infectious Diseases, Nanfang Hospital, Southern Medical University, Guangzhou, China

**Keywords:** hepatitis B virus (HBV), non-canonical NF-κB signaling, antiviral immunity, NF-κB-inducing kinase (NIK), p100/p52 (NFKB2)

## Abstract

The non-canonical nuclear factor kappa-light-chain-enhancer of activated B cells (NF-κB) signaling pathway is an important component of NF-κB transcription complex. Activation of this pathway mediates the development and function of host immune system involved in inflammation and viral infection. During hepatitis B virus (HBV) infection, there is a complex interaction between infected hepatocytes and the immune cells, which can hinder antiviral immune responses and is associated with pathological changes in liver tissue. Consistently, the host immune system is closely related to the severity of liver damage and the level of viral replication. Previous studies indicated that the non-canonical NF-κB signaling pathway was affected by HBV and might play an important regulatory role in the antiviral immunity. Therefore, systematically elucidating the interplay between HBV and non-canonical NF-κB signaling will contribute the discovery of more potential therapeutic targets and novel drugs to treat HBV infection.

## Introduction

**Figure 1:**
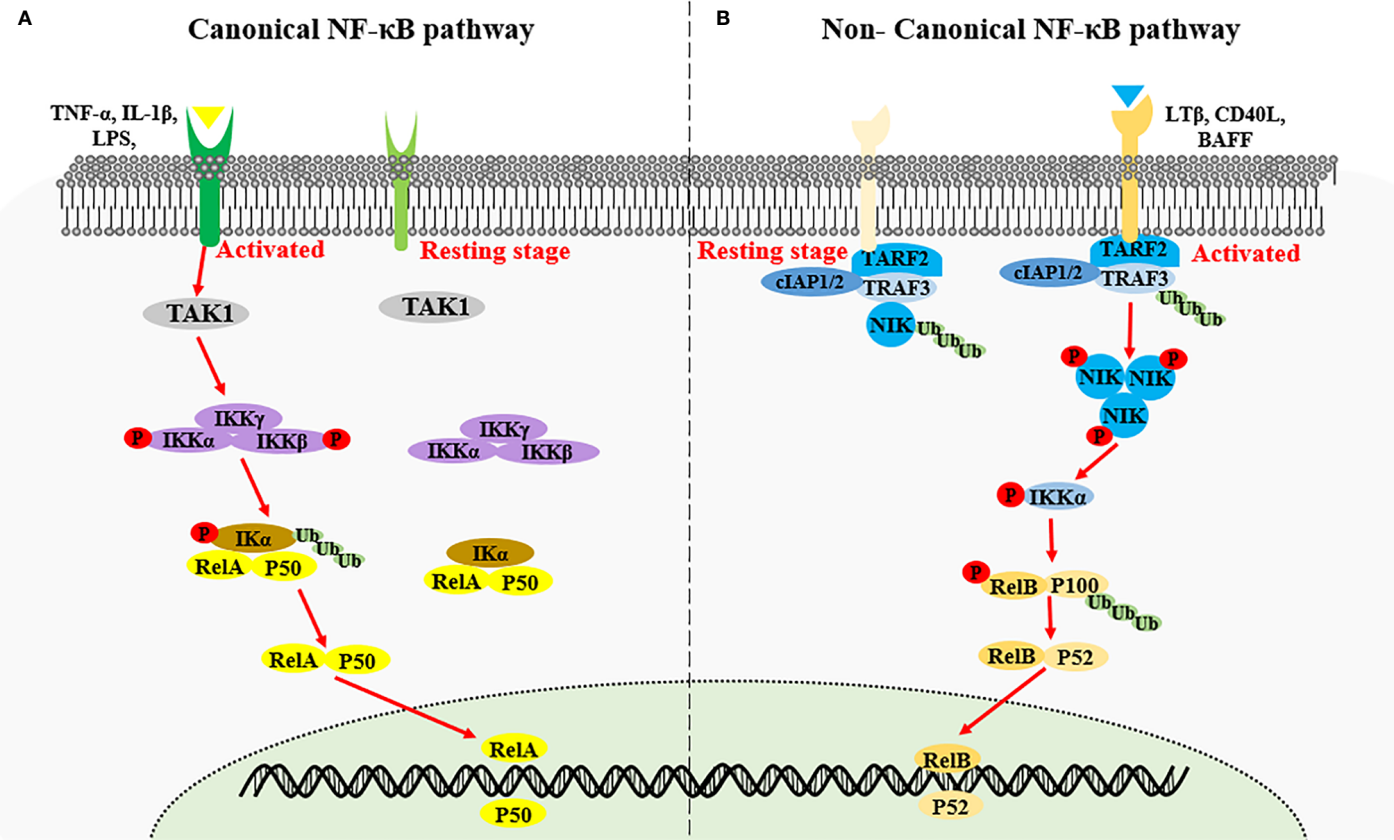
Activation of canonical NF-κB signaling and non-canonical NF-κB signaling. **(A)** Canonical NF-κB signaling is stimulated by proinflammatory cytokines such as IL-1β, TNF-α and LPS, inducing the activation of IKK complex by TAK1. The IKK complex then phosphorylates the IκB kinase α/β (IKKα/β), causing the ubiquitin-dependent degradation of κB inhibitory factors (IκBs) protein, thereby triggering the nuclear transcription factor heterodimer RelA/p50. **(B)** Non-canonical NF-κB signaling is activated by the specific TNFR superfamily. Receptor activation induce the recruitment of TRAF3-TRAF2-cIAP1/2 receptor complex, followed by the degradation of TRAF3 *via* ubiquitination, resulting in the stabilization and accumulation of NIK. NIK phosphorylates IKKα which in turn phosphorate p100, triggering the ubiquitination and degradation of p100 to generate p52 and the nuclear transduction of RelB-p52 heterodimer.

Nuclear factor kappa-light-chain-enhancer of activated B cells (NF-κB) encompasses an important family of transcription factors, can regulate the expression of multiple genes, and has been implicated in diverse biological processes including innate and adaptive immunity, inflammation, stress, immune cell development, and lymphatic organ formation (1, 2). Based on the components of the signaling cascade, NF-κB signaling pathways can be categorized as canonical or non-canonical (3). The canonical NF-κB pathway is rapid and transient, and mainly stimulated by proinflammatory cytokines such as IL-1β, tumor necrosis factor (TNF)-α, antigen ligands, and toll-like receptors (TLRs). Activation of the canonical NF-κB pathway relies on phosphorylation and ubiquitination of IκB kinase α/β (IKKα/β) causing the degradation of κB inhibitory factors (IκBs) protein, thereby countering inhibition of the nuclear transcription factor heterodimer RelA/p50 by IκBs (4, 5). The non-canonical NF-κB signaling pathway is slow and persistent, and generally activated by ligands of the TNF receptor superfamily, including lymphotoxin beta (LTB), CD40, OX40, RANK, TWEAK and B cell-activating factor (BAFF). These ligands stimulation induces NF-κB-inducing kinase (NIK, also called MAP3K14) stabilization and accumulation, results in IKKα phosphorylation. Activated IKKα subsequently phosphorylates p100, triggering K48 linked ubiquitination and degradation of p100, generating p52 (also known as NFKB2). The nuclear translocation of RelB-p52 heterodimer then initiates the expression of target genes (4, 5) (**Figure 1**). NIK is a key molecule for non-canonical NF-κB pathway activation, and this process requires both NIK expression and kinase activity (6, 7). Expression of human NIK-dominant inactivated mutant (NIKKA, KK429/430AA) results in the blockage of the non-canonical NF-κB pathway (8).

It was reported that the alymphoplasia (aly/aly) mice with loss-of-function mutation of NIK exhibited aberrant development of spleen architecture and lymph nodes, impaired B cell proliferation and maturation, no formation of germinal center or isotype antibody switching in antigen-specific immune responses, and disordered thymus structure, and autoimmune inflammation in multiple organs (9, 10). Moreover, the phenotype of NFKB2 KO mice was similar to that of the aly/aly mice, with defects in splenic architecture, lymph nodes, and peripheral B cell maturation, as well as impaired antibody responses (11, 12). The severe immunodeficiencies observed in these mice strains indicates that the non-canonical NF-κB signaling pathway plays an essential role in multiple important biological processes, including the initiation and regulation of B cell and T cell immunity (13).

Host antiviral immunity is an important defense mechanism with respect to preventing virus invasion, and effectively clearing viral infection. Many RNA and DNA viruses, can regulate the activation of the non-canonical NF-κB pathway *via* different adaptor proteins, leading to the regulation of antiviral immunity (14). In contrast, some viruses can evade antiviral immunity by affecting the non-canonical NF-κB pathway. For example, the RNA virus EV71 upregulated NIK, the key protein of the non-canonical NF-κB pathway, to promote the secretion of inflammatory cytokines and apoptosis of EV71-infected cells (14, 15). Epstein-Barr virus, human T-lymphotropic virus 1, and Kaposi’s sarcoma associated herpesvirus could activate the non-canonical NF-κB pathway by encoding corresponding oncogenic proteins (16–18). Currently, increasing evidence suggests that the non-canonical NF-κB pathway also plays roles in host responses to hepatitis B virus (HBV) infection. In this review, we discussed the interplay between HBV and the non-canonical NF-κB pathway.

## Regulation of Non-Canonical NF-κB Signaling Pathway by HBV Infection

HBV is a DNA virus that encodes multiple proteins that affect the activation of the non-canonical NF-κB pathway, thus enabling regulation of the host defense response. The type I interferon (IFN) system, an indispensable part of the innate immune response to HBV, is involved in an immediate antiviral response *via* the induction of numerous functional proteins against the viral life cycle, and activates the adaptive immune response (19). TBK1 can function as a positive and negative regulator of the non-canonical NF-κB pathway (20). HBV polymerase (Pol) can evidently inhibit the phosphorylation, dimerization, and nuclear translocation of IRF3, as well as IFN-β expression at the TBK1/IKKƐ level (19). Activating NIK promote phosphorylation of IRF3 to produce type I interferon (21, 22). Therefore, the mechanism by which HBV pol protein inhibits IFN-β production in human hepatocytes may also be associated with the level of non-canonical NF-κB pathway activation. Liu et al. (23) reported that Pol protein did not alter the level of NF-κB expression, but could prevent the activation of the non-canonical NF-κB pathway required for IFN-β activation by inhibiting the nuclear translocation of RelB/p52 and NF-κB subunits in hepatoma cells. These studies suggested that Pol protein could inhibit IFN-β production, which may be associated with the non-canonical NF-κB pathway in hepatocytes. However, additional studies showed some conflicting results. As observed in chronic HBV infection patients, the IFN-stimulated genes (ISGs) were not activated in liver tissues (24). In addition, results of the *in vitro* models of HBV-infected hepatocytes also found that HBV infection did not induce type I and III IFNs, and the downstream ISGs (25, 26), which further supported the inability of HBV to induce IFN response. Thus, the relationship between Pol protein, IFN signaling and non-canonical NF-κB pathway remains to be explored.

HBx protein plays a vital role in HBV replication, and is a potential inducer for hepatocellular carcinoma development (HCC) (27, 28). Previous studies indicate that HBx induced the phosphorylation and degradation of intracellular IkBα to activate the classical NF-κB pathway, accompanied with slight decrease in the level of p100 protein, suggesting that HBx may be involved in activation of the non-canonical NF-κB pathway (29, 30). Kim et al. (31) reported that HBx might disturb the activation of the non-canonical NF-κB pathway *via* a NIK-IKK-IkB-dependent mechanism in the liver, and result in the induction of inflammatory response or hepatic oncogenesis by stimulating hepatocyte proliferation. But whether the effect mediated by HBx is valid in the HBV infection need to be further studied.

HBV e antigen (HBeAg), a secreted protein and not required for viral replication, is thought to play an immunoregulatory role and promote viral persistence during viral infection. Researches by Wang et al. (32) has found that HBeAg can associate with NEMO, the regulatory subunit for IκB kinase (IKK) that controls the NF-κB signaling pathway, and thereby inhibited TRAF6-mediated K63-linked ubiquitination of NEMO induced by IL-1β, resulting in the downregulation of NF-κB activity and increased virus replication. HBeAg suppresses LPS-induced NLRP3 inflammasome activation, pro-IL-1β expression and IL-1β production in kuffer cells not only *via* inhibiting NF-κB phosphorylation but inhibiting ROS production (33). The roles of HBeAg in regulating the NF-κB pathway to affect host immune response are controversial during the infection of HBV. In addition, the HBeAg affected NEMO mainly functions in canonical NF-κB signaling pathway. Nevertheless, canonical and non-canonical NF-κB activation pathways are connected *via* many crosstalk mechanisms. Therefore, HBeAg affecting the host immune response also cannot exclude the role of non-canonical NF-κB pathway.

## Regulation of HBV Infection by Non-Canonical NF-κB Signaling Pathway

**Figure 2:**
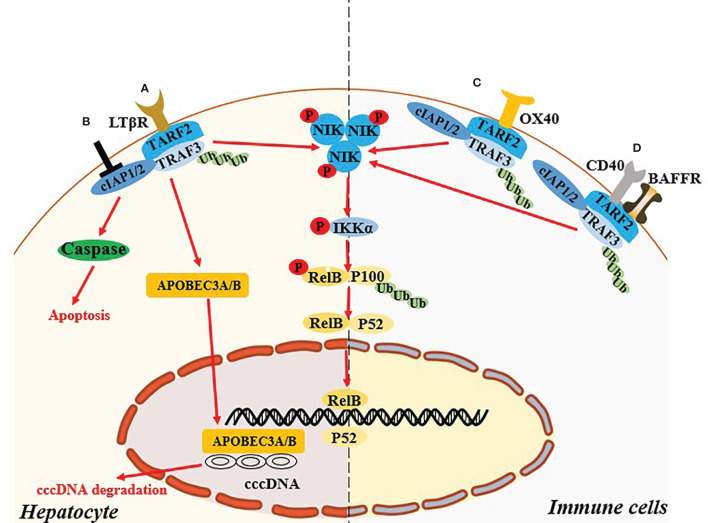
Non-canonical NF-κB signaling pathway regulates the HBV infection. **(A)** In hepatocyte, activation of LTB receptor in hepatocyte mediates the non-canonical NF-κB signaling pathway and induce the up-regulated expression of APOBCE3A/B protein, which degrade cccDNA. **(B)** Targeting cIAPs also mediate the activating the NIK-dependent non-canonical NF-κB signaling pathway and exert the antiviral effect in hepatocyte *via* TNF-mediated death of infected cells. **(C, D)** Specific ligation of OX40, BAFFR or CD40 in immune cells might recover the exhausted immune function during HBV infection through the activation of the NIK-dependent non-canonical NF-κB signaling pathway.

Abnormal host immune function and continuous HBV replication are the main causes of disease progression in patients with chronic hepatitis B (CHB) (34). Concurrent continuous HBV replication and the activation of inflammatory pathways lead to chronic liver damage (35). Immune system is very important for the elimination of HBV, but CHB patients tend to exhibit defective innate and adaptive immune function, and cannot effectively remove viral products in the liver. Previous studies indicated that non-classical NF-κB pathway in the hepatocytes and immune cells played an important role in control of HBV replication *in vitro* and clearance of HBV *in vivo* (**Figure 2**).

### Hepatocytes-Intrinsic Non-Canonical NF-κB Signaling Pathway

HBV replication in hepatocytes may be related to NIK-dependent activation of the non-classical NF-κB pathway. Microarray analysis and western blotting validation suggested that mRNA and protein levels of TRAF2 and NIK in primary hepatocytes are upregulated during the early stage of HBV infection (36). Lymphotoxin beta receptor, a member of the TNF receptor superfamily, is able to activate the NIK-dependent non-canonical NF-κB pathway. Activation of this receptor could induce the expression of APOBEC3A/3B protein, which mediated deamination on the negative chain of cccDNA to degrade it (37). The above results suggested that the non-canonical NF-κB pathway activation in hepatocytes might exert a direct anti-HBV effect.

Cellular inhibitor of apoptosis proteins (cIAPs) are a family of highly conserved endogenous anti-apoptotic molecules (38). In the resting state, cIAP1/2 uses TRAF2 as an adaptor protein to connect with NIK binding protein TRAF3 to form a TRAF3-TRAF2-cIAP1/2 multi-subunit ubiquitin ligase complex, resulting in very low protein levels of cellular endogenous NIK (39). Degradation of cIAPs or loss of TRAF2 can continuously activate the NIK-dependent non-canonical NF-κB signaling pathway (39). Ebert et al. (40) reported that cIAPs can impair viral clearance by preventing TNF-mediated death of infected cells, and mice with hepatocyte-specific deficiency of cIAPs expression promoted the clearance chronic HBV infection. Moreover, birinapant, a chemical inhibitor of cIAPs, could reduce HBV DNA and hepatitis B surface antigen levels in serum, and induce hepatitis B core antigen-positive hepatocyte apoptosis in HBV persistence mouse model (41). Therefore, whether the antiviral effect mediated by targeting cIAPs is related to activation of the NIK-mediated non-canonical NF-κB signaling pathway need further investigation.

### Immune Cells-Intrinsic Non-Canonical NF- κB Signaling Pathway

Abnormal expression and function of specific ligands and receptors involved in activation of the non-classical NF-κB pathway in the immune cells might be related to the chronicity of HBV infection. OX40 (also known as CD134) is an important costimulatory molecule in T cells, and its ligand OX40L (also known as CD252) is mainly expressed on antigen-presenting cells. OX40 ligation can promote T cell phenotypic transformation, maintain the activation state, and promote the proliferation of effector T cells and memory T cells, while also inhibiting the differentiation and activity of regulatory T cells (Tregs) (42, 43). Interestingly, studies suggest that the key to determining antiviral immunity is the expression of the costimulatory molecule OX40L on hepatic innate immune cells, and the expression of OX40L is positively correlated with age (44). OX40 agonists can increase the HBV clearance rate in a young mouse model of hepatitis B, as well as the strength of T cell responses in young mice and adult mice that were exposed to HBV when they were young and developed a CHB serological profile (44). Moreover, in adult humans HBV clearance is associated with increased OX40 expression in peripheral CD4^+^ T cells (44). In a recent study OX40 was highly expressed on CD4^+^ T cells in the immune microenvironment of HBV-related HCC (45). OX40 agonists combined with PD-1 blockers can enhance the function of HBV-specific CD4^+^ T cells (42). The above studies suggest that in innate immune cells and T cells the OX40-mediated non-canonical NF-κB pathway may participate in HBV clearance by affecting the function of immune cells. In a liver injury model in HBsAg transgenic mice mediated by natural killer (NK) cells induced with low or high doses of concanavalin A, the proportion of CD4^+^CD25^+^FoxP3^+^ Tregs in the liver increases, and the proportion of restored Tregs continued to increase with the development of liver damage (46). The mechanism of reducing liver injury in HBs transgenic mice is related to the direct inhibition of NK cell-mediated hepatotoxicity by Tregs *via* OX40/OX40L interaction in cell-cell contact (46). In patients with CHB the immune microenvironment is characterized by the exhaustion of virus-specific T cells and an increase in negatively regulated immune cells such as Tregs. Therefore, this may also be related to the non-classical NF-κB pathway regulated by OX40/OX40L.

BAFF is an essential cytokine for the activation of B lymphocytes, and BAFF receptor is expressed on B cells (47). Interaction between BAFF and BAFF receptor can also activate the non-canonical NF-κB signaling pathway, and its mediated activation plays an important role in regulating the survival and maturation of peripheral B cells (48). Studies indicate that in CHB patients’ serum BAFF is maintained at a high level, leading to excessive activation of B cells, thus changing T cell functions (upregulating PD-1, Tim-3, and Lag-3, *i.e.*, exhaustion phenotypes) and reducing reactions to PEG-IFN (49, 50). Notably however, the mechanisms by which BAFF mediates the chronicity of HBV require further clarification. Studies suggest that *in vitro* administration of CD40L signals can partially restore the function of hepatitis B surface antigen (HBsAg) -specific B cells (51). CD40-CD40L interaction can induce activation of the non-canonical NF-κB pathway in mouse splenic B cells (52). The above results suggest that non-canonical NF-κB pathway dysregulation in immune cells of patients with CHB may be related to the failure of HBV clearance and disease progression.

NIK is a key adaptor protein associated with activation of the non-classical NF-κB pathway, and may also participate in the development of CHB disease by affecting the function of immune cells. In previous studies NIK knockout in dendritic cells affected antigen presentation to CD8^+^ T cells (53). T cell-specific NIK deficiency induces naive T cells to differentiate into effector T cells and memory T cells (54, 55). Moreover, in T cell-specific NIK-deficient mice the phosphorylation of Zap70, LAT, AKT, ERK1/2, and PLCγ is hindered, thereby blocking the T cell receptor signaling pathway (56). The continuous expression of viral antigens and the hepatic inflammatory microenvironment can downregulate the function and proportion of HBV-specific CD4^+^ cells, CD8^+^ T cells, dendritic cells, NK cells, and NK T cells in CHB patients. Therefore, the exhaustion of immune function in CHB patients may be related to the non-canonical NF-κB pathway dysregulation caused by downregulation of NIK in immune cells such as dendritic cells and T cells. Whether the inability of antiviral immunity in CHB patients is related to the activation of the non-canonical NF-κB pathway in these immune cells remains to be determined. In addition, IFN-γ and TNF-α produced by T cells can degrade the cccDNA through deamination, but not the cytolysis in hepatocyte, suggesting that the non-classical NF-κB pathway has a complex regulatory network in hosts with normal immunity (57).

## Conclusion

To date studies indicate that the non-canonical NF-κB signaling pathway plays an important role in the development of the immune system and triggering inflammation, but research on the regulatory role of the HBV life cycle and host antiviral effects remains largely unknown. A variety of ligands and receptors, adaptor proteins, and regulatory factors involved in the formation of non-canonical NF-κB signaling pathway might play important roles in the establishment and development of HBV disease. Therefore, in-depth mechanistic studies investigating the interplay between HBV and non-canonical NF-κB signaling may provide potential strategies for HBV treatment.

## Author Contributions

XZ, XL, QC, and HL collected the data and drafted the manuscript. XZ made critical revision of the manuscript for important intellectual content. All authors contributed to the article and approved the submitted version.

## Funding

This review was partly supported by grants from the National Natural Science Foundation of China (No. 81871664 and No.81970539).

## Conflict of Interest

The authors declare that the research was conducted in the absence of any commercial or financial relationships that could be construed as a potential conflict of interest.

## Publisher’s Note

All claims expressed in this article are solely those of the authors and do not necessarily represent those of their affiliated organizations, or those of the publisher, the editors and the reviewers. Any product that may be evaluated in this article, or claim that may be made by its manufacturer, is not guaranteed or endorsed by the publisher.
